# Advanced glycation end-products reduce podocyte adhesion by activating the renin-angiotensin system and increasing integrin-linked kinase

**DOI:** 10.3892/etm.2013.1312

**Published:** 2013-09-23

**Authors:** CAILIAN CHENG, ZHENDA ZHENG, CHENGGANG SHI, XUN LIU, ZENGCHUN YE, TANQI LOU

**Affiliations:** 1 Department of Nephrology, The Third Affiliated Hospital of Sun Yat-sen University, Guangzhou, Guangdong 510630, P.R. China; 2 Department of Cardiology, The Third Affiliated Hospital of Sun Yat-sen University, Guangzhou, Guangdong 510630, P.R. China

**Keywords:** advanced glycation end-products, podocyte, adhesion, integrin-linked kinase

## Abstract

The aim of this study was to investigate the effects of advanced glycation end-products (AGEs) on podocyte adhesion and the underlying mechanisms. Immortalized mouse podocytes were exposed to various conditions and podocyte adhesion was evaluated using a hexosaminidase assay. The expression levels of integrin-linked kinase (ILK) were measured by quantitative polymerase chain reaction (qPCR) and western blotting. Treatment with AGEs resulted in a significant, concentration-dependent reduction in podocyte adhesion (P<0.05) and an incremental rise in ILK expression up to a maximum of 100%. Pretreatment with losartan significantly prevented the upregulation of ILK and attenuated the loss of podocyte adhesion observed in podocytes exposed to AGEs (P<0.05). However, the adhesion of losartan-treated podocytes remained lower than that of the podocytes exposed to bovine serum albumin. The results indicate that AGEs reduce podocyte adhesion via the upregulation of ILK expression, which occurs partly through activation of the renin-angiotensin system in podocytes.

## Introduction

Diabetic nephropathy (DN) is the leading cause of chronic and end-stage renal disease. It is responsible for significant morbidity and mortality in patients with diabetes. The production of advanced glycation end-products (AGEs) is accelerated in diabetes and AGEs mediate the progressive alteration of renal architecture and loss of renal function in DN (1).

The clinical signature of DN is proteinuria, which is a marker of disease severity and an independent risk factor for cardiovascular disease. Podocytes are the most differentiated cell type within the glomerulus and are crucial for the maintenance of the glomerular filtration barrier. Experimental and clinical studies have shown that podocyte numbers are decreased in DN, and there is a direct correlation between decreased podocyte number and proteinuria (2). Moreover, many podocytes in urine are viable, suggesting a primary problem was their ability to remain attached to the underlying glomerular basement membrane (GBM) (3). A reduction in podocyte number may result in the failure of mechanical support for the glomerular capillary loop, leading directly to glomeruloscerosis (4).

Integrin-linked kinase (ILK) is important for the control of podocyte-matrix adhesion and shape modulation. Treatment of podocytes with the ILK inhibitor MC-5 has been shown to reduce stress fiber formation and increase apoptosis (5). However, little is known with regard to the effects of AGEs on ILK expression and podocyte adhesion.

It is increasingly being suggested that sustained intrarenal renin-angiotensin system (RAS) activation is crucial in the pathogenesis of podocyte injury and causes proteinuria. The blockade of the RAS with angiotensin-converting enzyme inhibitors and angiotensin II type 1 receptor (AT1R) antagonists has been shown to reduce proteinuria and retard DN progression (6). These observations have indicated that RAS blockade may directly affect various renal cells. Previous studies have demonstrated that blocking the RAS restored nephrin levels and attenuated foot process broadening in DN (7,8). The inhibition of angiotensin II (Ang II) may directly benefit podocytes. Our previous study (9) showed that AGEs activated the RAS in podocytes. Accordingly, the current study investigated whether the activated RAS was involved in the reduction of podocyte adhesion induced by AGEs.

## Materials and methods

### Cell culture

A conditionally immortalized mouse podocyte cell line was provided by Dr Peter Mundel (Harvard Medical School, Charlestown, MA, USA). Cells were harvested as described in our previous study (9) and cultured in nonpermissive conditions in 10% fetal bovine serum (FBS) with 10 U/ml interferon (INF)-γ (Sigma-Aldrich, St. Louis, MO, USA) in RPMI-1640 medium at 33°C. When the podocytes were 60–80% confluent, the temperature was changed to 37°C and incubation was continued for 7–10 days to allow differentiation. Passages 8–14 were used for all experiments; prior to treatment, differentiated podocytes were cultured in media containing 1% FBS for 24 h. Bovine serum albumin (BSA) was purchased from MP Biomedicals (Santa Ana, CA, USA), while AGEs were obtained from Merck (Darmstadt, Germany) and losartan was from Sigma-Aldrich.

### Experimental design

Growth-arrested podocytes were exposed to different concentrations of AGEs (20, 40, 80 and 160 μg/ml) for 24 h prior to cell adhesion, ILK mRNA and protein expression being measured. Growth-arrested podocytes were pretreated for 1 h with or without losartan (100 μM) and AGEs were subsequently added.

### RNA extraction, cDNA synthesis and quantitative polymerase chain reaction (qPCR)

Total RNA was extracted from the podocytes using TRIzol reagent (Invitrogen Life Technologies, Carlsbad, CA, USA), according to the manufacturer’s instructions. Total RNA (500 ng) was reverse transcribed using reverse transcriptase (RT) in a SYBR Premix Ex Taq kit (Perfect Real-Time; Takara Bio, Inc., Shiga, Japan). Reactions were carried out at 37°C for 15 min and then 85°C for 5 sec. qPCR was performed using an ABI Prism 7000 sequence detection system (Applied Biosystems, Foster City, CA, USA) and a SYBR Premix Ex Taq Perfect Real-Time kit (Takara Bio, Inc.). The primers used were as follows: ILK sense, GAC GCT CAG CAG ACA TGT GGA and anti-sense, GGA AAT ACC TGG TGG GAC GGT AG; glyceraldehyde-3-phosphate dehydrogenase (GAPDH) sense, AAA TGG TGA AGG TCG GTG TGA AC and anti-sense, CAA CAA TCT CCA CTT TGC CAC TG.

### Western blot analysis

Podocytes were washed twice with cold phosphate-buffered saline (PBS) and scraped with lysis buffer containing 20 mM Tris-HCl (pH 7.5), 150 mM NaCl, 1 nM Na_2_EDTA, 1 mM EGTA, 1% Triton, 2.5 mM sodium pyrophosphate, 1 mM β-glycerophosphate, 1 mM Na_3_VO_4_, 1 μg/ml leupeptin (Cell Signaling Technology, Inc., Danvers, MA, USA) and protease and phosphatase inhibitor cocktail tablets (Roche Diagnostics, Mannheim, Germany). Protein concentrations were determined using the Bradford reaction. From boiled extracts, 20 μg was loaded on 8% sodium dodecyl sulfate-polyacrylamide gel electrophoresis (SDS-PAGE) gels and transferred to polyvinylidene fluoride membranes (Bio-Rad Laboratories, Hercules, CA, USA). Membranes were blocked in 5% fat-free milk prior to incubation with rabbit anti-ILK (1:500; Santa Cruz Biotechnology, Inc., Santa Cruz, CA, USA). Blots were incubated with horseradish peroxidase-conjugated secondary antibodies (Santa Cruz Biotechnology, Inc.); bands were detected using an enhanced chemiluminescence (ECL) system (Millipore, Billerica, MA, USA).

### Cell adhesion assay

Podocytes were harvested, washed, and resuspended in medium with different concentrations of AGEs. Equal numbers of cells (1×10^5^) were replated in duplicate wells of 96-well plates coated with rat-tail type I collagen (Sigma, St. Louis, MO, USA) and allowed to attach at 37°C in a CO_2_ incubator for 6 h. Nonattached cells were removed by washing with PBS twice. Attached cells were measured using a hexosaminidase assay, as described in a previous study (5). Briefly, 3.75 mM *p*-nitrophenol-N-acetyl-D-glucosaminide (Sigma) in 50 mM citrate buffer (pH 5.0) containing 0.25% Triton X-100 was added to each well for 1 h at 37°C. Enzyme deactivation was performed using 50 mM glycine and 5 mM EDTA (pH 10.4). Cell adhesion was quantified using absorbance at 405 nm, measured with a Bioteck Spectramax (Molecular Devices, Sunnyvale, CA, USA). All data were corrected with values from control wells and experiments were repeated three times.

### Statistical analyses

Results are presented as the mean ± standard deviation for different conditions. Statistical significance was assessed using a nonparametric Kruskal-Wallis analysis of variance (ANOVA) or a Student’s t-test. P<0.05 was considered to indicate a statistically significant difference.

## Results

### AGEs decrease podocyte adhesion

**Figure 1:**
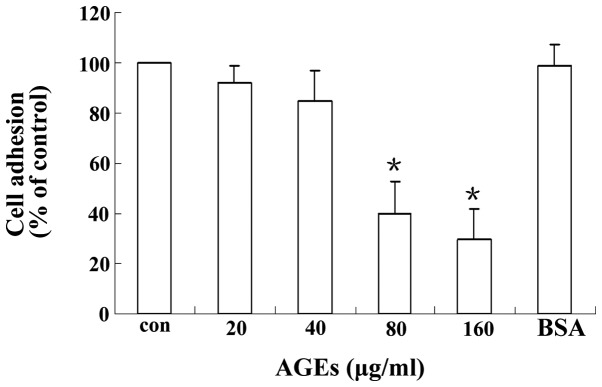
Advanced glycation end-products (AGEs) reduce podocyte adhesion. Podocytes were incubated with AGEs for 24 h. Adhesion was measured using a hexosaminidase assay. AGEs concentration was measured in μg/ml; bovine serum albumin (BSA), 80 μg/ml. Cell adhesion was normalized to the control (con) group. ^*^P<0.05 vs. the con group. con, an untreated control group.

Podocytes were incubated with different concentrations of AGEs for 24 h and podocyte adhesion was measured using a hexosaminidase assay. As shown in Fig. 1, AGEs inhibited podocyte adhesion in a concentration-dependent manner. Podocytes incubated with AGEs (80 or 160 μg/ml) showed significantly inhibited adhesion compared with cells in the control group: for 80 μg/ml, 40±13 versus 100%; for 160 μg/ml, 30±12 versus 100% (P<0.05).

### AGEs upregulate ILK mRNA and protein levels in podocytes

**Figure 2:**
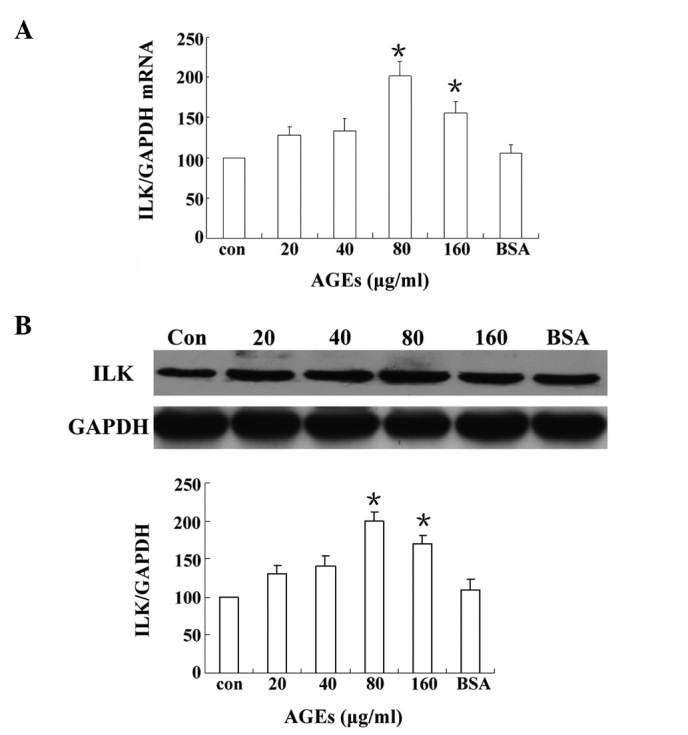
Advanced glycation end-products (AGEs) increase integrin-linked kinase (ILK) mRNA and protein expression in podocytes. Podocytes were treated with AGEs for 24 h. Untreated podocytes (con) and podocytes treated with bovine serum albumin (BSA; 80 μg/ml) were used as the controls. (A) ILK mRNA was assayed by quantitative polymerase chain reaction (qPCR). (B) Lysates were immunoblotted for ILK and housekeeping proteins. AGEs concentration was measured in μg/ml; BSA, 80 μg/ml BSA. ^*^P<0.05 vs. the con group. GAPDH, glyceraldehyde-3-phosphate dehydrogenase.

To investigate the potential role of ILK in podocyte adhesion, the effects of different concentrations of AGEs on ILK expression in podocytes were observed. As shown in Fig. 2, ILK mRNA and protein production were rapidly upregulated by AGEs in a concentration-dependent manner. Concentration-response studies revealed that the maximal expression of ILK was induced by AGEs at a concentration of 80 μg/ml. Moreover, ILK expression levels in mouse podocytes exposed to 80 μg/ml AGEs were two-fold higher than the expression in the control cells (Fig. 2B). A further increase in the concentration of AGEs beyond 80 μg/ml did not result in additional induction of ILK.

### Preincubation with losartan improves the adhesion of AGEs-treated podocytes

**Figure 3:**
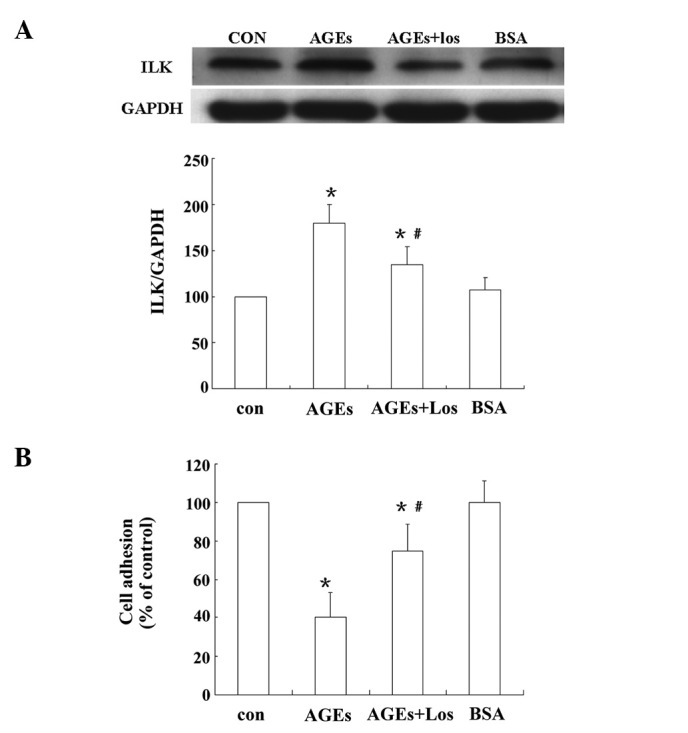
Losartan (Los) prevents the increase in integrin-linked kinase (ILK) expression and improves the adhesion of podocytes treated with advanced glycation end-products (AGEs). Podocytes were pretreated with Los (100 μM) for 60 min prior to AGEs treatment. Lysates were immunoblotted for ILK and housekeeping proteins, while podocyte adhesion was evaluated using a hexosaminidase assay. (A) Los prevented the increase in ILK levels induced by the AGEs. (B) Los improved the adhesion of podocytes treated with AGEs. Results are presented as the mean ± standard deviation for three independent experiments. ^*^P<0.05 vs. control (con) group; ^#^P<0.05 vs. AGEs. GAPDH, glyceraldehyde-3-phosphate dehydrogenase; AGEs, 80 μg/ml; AGEs + Los, 80 μg/ml AGEs + 100 μM Los; bovine serum albumin (BSA), 80 μg/ml BSA. con, an untreated control group.

The effects of the angiotensin II receptor blocker losartan on the adhesion of AGEs-treated podocytes were analyzed. As shown in Fig. 3A, AGEs increased ILK expression significantly; however, the AGE-induced ILK expression was significantly inhibited by 48% following pretreatment with losartan (P<0.05). A cell adhesion assay revealed that, compared with control cells, adhesion was significantly inhibited when podocytes were exposed to 80 μg/ml AGEs (40±13 versus 100%; P<0.05). Pretreatment with losartan (100 μM) significantly improved the podocyte adhesion compared with that of the AGEs-treated cells (75±13 versus 40±13%; P<0.05; Fig. 3B).

## Discussion

Podocytes are a monolayer of cells on the urinary side of the GBM; a critical number of podocytes is required for normal functions. Focal areas in the glomerulus where the podocyte number is reduced are vulnerable to protein loss (10). Podocytopenia is associated with the development of glomerular sclerosis and loss of renal function, which result from podocyte detachment, apoptosis and inability to proliferate (10). A number of studies (4,11) have shown that the majority of urinary podocytes are viable and are able to attach to collagen-coated plates. This suggests that the primary podocyte problem in diabetes is the inhibition of cell-matrix interactions rather than cell death. Moreover, loss of cell contact with the GBM may trigger apoptotic death. In DN, urinary podocyte loss is a more specific marker of ongoing glomerular damage than proteinuria (12). Furthermore, podocyte depletion is a major mechanism driving glomeruosclerosis. When podocyte depletion reaches a threshold (~30%), it activates continuous autonomous podocyte depletion until global depletion occurs (13).

ILK has been implicated in the regulation of numerous aspects of cellular signaling, including integrin activation, fibronectin matrix assembly, survival and differentiation. ILK interacts with the cytoplasmic domains of β1 and β3 integrins and mediates integrin signaling, which participates in podocyte adhesion. However, the effects of AGEs on ILK expression have not yet been elucidated. The results of the current study indicated that AGEs decreased the adhesive capacity of podocytes in a concentration-dependent manner. In addition, ILK expression was upregulated significantly in response to AGEs. Upregulation of ILK expression is a common response of podocytes to injury and represents a convergent pathway that mediates podocyte dysfunction and proteinuria. A previous study (14) demonstrated that the upregulation of ILK expression in diabetic rats was positively correlated with podocyte injury and albuminuria. ILK is essential in podocyte biology. Aberrant regulation of ILK has been shown to induce shrinkage of the podocyte cell body and elongation of the podocyte processes (15) and is implicated in the pathogenesis of epithelial-mesenchymal transition, an explanation for the detachment of podocytes from the GBM (16).

The activation of the RAS is important in progressive DN. Ang II, the final effector of the RAS, is crucial in the pathogenesis of DN, particularly in podocyte injury. Our previous study (9) revealed that Ang II levels in conditioned media and cell lysates increased significantly in podocytes exposed to AGEs compared with the levels in a control group. To investigate whether the local RAS was involved in the adhesion of podocytes treated with AGEs in the present study, podocytes were pretreated with the AT1R antagonist losartan. The results of the current study showed that pretreatment with losartan significantly improved podocyte adhesion and decreased ILK levels incrementally and significantly. These results indicated that the RAS activation induced by AGEs was involved in the inhibition of podocyte adhesion. A previous study (14) revealed that the inhibition of ILK ameliorated the dysfunction of podocyte adhesion. Other studies demonstrated that blocking the RAS reduced the progression of proteinuria and nephropathy and that this effect was not explained solely by an antihypertensive effect. The protective effect of Ang II receptor blockade has been shown to be entirely accounted for by a reduction in podocyte loss (13,17). These observations suggest that RAS blockade directly affects podocytes. Podocytes have been a focus of study, due to the fact that they are a filtration barrier to proteins and are important in the pathogenesis of glomerulosclerosis (18). Liebau *et al*(19) first demonstrated the functional expression of key RAS components in differentiated human podocytes using western blot analysis and immunostaining (19). Ang II is emerging as a critical mediator of podocyte injury in diabetic kidney disease and has been shown to cause the rearrangement of cortical F-actin, redistribution of ZO-1, reduced α-actinin-4, dephosphorylation of nephrin, expression of focal adhesion kinase and a migratory phenotype switch in cultured mouse podocytes (20,21). The results of the present study indicate that AGEs may reduce podocyte adhesion through the upregulation of ILK expression, and that local RAS activation in podocytes may be important in the process.

The results of the present study indicated that the AGEs-induced RAS activation in podocytes is important in the pathophysiology of podocyte depletion, particularly in podocyte adhesion. A previous study showed that collagen modified by AGEs inhibited podocyte adhesion (22), while another study demonstrated that AGEs inhibit podocyte adhesion via the suppression of neuropilin 1 (NRP1) expression (23). The findings of the present study indicate that AGEs directly inhibit the adhesive capacity of podocytes via the upregulation of ILK expression and the activation of the local RAS. However, it was not possible to restore the podocyte adhesive capacity, even when the podocytes were pretreated with losartan, which may be due to the fact that AGEs have other effects on podocytes, similar to those of transforming growth factor-β1 and heparanase, which decrease the podocyte adhesive capacity (24,25). These effects may be responsible for podocyte damage and continuous loss in DN. The present study had certain limitations, such as only using a conditionally immortalized mouse podocyte cell line and a single inhibitor of ILK, losartan. Whether these results may be extended to primarily cultured podocytes and *in vivo* conditions, such as animal models of DN induced by streptozotocin, remains to be determined. However, the current data indicate that AGEs decreased podocyte adhesion via the upregulation of ILK, and that angiotensin receptor blockers may be an attractive therapeutic strategy for preventing the pathogenic effect of AGEs.

In conclusion, the results of this study indicated that AGEs inhibited podocyte adhesive capacity through RAS activation and the upregulation of ILK synthesis. These results provide important information for the largely unknown mechanism of AGEs-mediated podocyte damage in DN. The elucidation of further details in this signaling pathway is likely be useful for future DN treatment choices.
